# Bringing CLARITY to the human brain: visualization of Lewy pathology in three dimensions

**DOI:** 10.1111/nan.12293

**Published:** 2015-12-07

**Authors:** A. K. L. Liu, M. E. D. Hurry, O.T. W. Ng, J. DeFelice, H. M. Lai, R. K. B. Pearce, G. T‐C. Wong, R. C‐C. Chang, S. M. Gentleman

**Affiliations:** ^1^ Neuropathology UnitDivision of Brain SciencesDepartment of MedicineImperial College LondonLondonUK; ^2^ Laboratory of Neurodegenerative DiseasesSchool of Biomedical SciencesLKS Faculty of MedicineThe University of Hong KongPokfulamHong Kong; ^3^ Department of AnaesthesiologyLKS Faculty of MedicineThe University of Hong KongPokfulamHong Kong; ^4^ State Key Laboratory of Brain and Cognitive SciencesThe University of Hong KongPokfulamHong Kong; ^5^ Research Centre of Heart, Brain, Hormone, and Healthy AgingLKS Faculty of MedicineThe University of Hong KongPokfulamHong Kong

**Keywords:** CLARITY, human *post mortem* brain, Lewy body pathology, three‐dimensional visualization, tissue clearing

## Abstract

**Aims:**

CLARITY is a novel technique which enables three‐dimensional visualization of immunostained tissue for the study of circuitry and spatial interactions between cells and molecules in the brain. In this study, we aimed to compare methodological differences in the application of CLARITY between rodent and large human *post mortem* brain samples. In addition, we aimed to investigate if this technique could be used to visualize Lewy pathology in a *post mortem* Parkinson's brain.

**Methods:**

Rodent and human brain samples were clarified and immunostained using the passive version of the CLARITY technique. Samples were then immersed in different refractive index matching media before mounting and visualizing under a confocal microscope.

**Results:**

We found that tissue clearing speed using passive CLARITY differs according to species (human *vs*. rodents), brain region and degree of fixation (fresh *vs*. formalin‐fixed tissues). Furthermore, there were advantages to using specific refractive index matching media. We have applied this technique and have successfully visualized Lewy body inclusions in three dimensions within the nucleus basalis of Meynert, and the spatial relationship between monoaminergic fibres and Lewy pathologies among nigrostriatal fibres in the midbrain without the need for physical serial sectioning of brain tissue.

**Conclusions:**

The effective use of CLARITY on large samples of human tissue opens up many potential avenues for detailed pathological and morphological studies.

## Introduction

Interest in studying the brain in fine molecular detail extends as far back as the 19^th^ century where Ramón y Cajal adapted the Golgi staining method of silver impregnation to visualize different classes of neurones. This provided the first evidence to suggest that neural networks are organized based on connectivity and coincided with the early development of light microscopy 1. Subsequently, confocal microscopy with optical sectioning capability has been developed to allow visualization of thick tissue in high resolution 2. This can be used with a combination of fluorescently labelled samples for the study of structural connectomics. However, tissue samples have to be cleared to prevent light scattering from lipids in brain tissues.

The earliest detailed account of tissue clearing was documented by the German anatomist Werner Spalteholz in 1911, who used wintergreen oil (methyl Salicylate) and benzyl benzoate to render heart tissue transparent to visualize the vasculature of the heart 3. The Spalteholz technique is still used today to visualize deep blood vessels, e.g. hippocampal vessels 4, but it has the disadvantage of causing severe tissue damage after long‐term incubation (over 5 months) with various organic solvents. Subsequently, the use of benzyl alcohol–benzyl benzoate (BABB/Murray's clear) clearing strategy 5, 6 and 3DISCO 7, 8 (Table 1) were developed from this technique in combination with fluorescently tagged samples for the three‐dimensional visualization of rodent brain structures. However, major drawbacks of using organic solvents in these techniques are the rapid quenching of fluorescence signals and the incomplete clearance of heavily myelinated structures. Aqueous‐based brain clearing strategies such as Sca*l*e 9, SeeDB 10, ClearT 11 and CUBIC 12 have been developed (Table 1). However, other problems such as protein loss, poor transparency, tissue swelling and lack of molecular probing capability remain an issue.

**Table 1:** Comparison of different tissue clearing techniques

Reference	Method	Principle and agent for clearing	Clearing time	Storage time	Tissue size change	IHC compatibility	Reprobing capability	Tracer compatibility	Method applied on human brain?
Spalteholz (1911) 3	Spalteholz's technique	Ethanol and benzene to dehydrate tissue. Wintergreen oil (methyl salicylate) and benzyl benzoate (1:1) for refractive index matching	From dehydrating to clearing ~5 months	Many months	Decrease	No	No	No	Yes
Dodt *et al*. (2007) 5	BABB/Murray's clear	Ethanol and hexane to dehydrate tissue. 1 part benzyl alcohol to 2 parts benzyl benzoate for refractive index matching	~10 days (whole mouse brain <2 weeks old) ~1.5 days (mouse hippocampus)	Maximum of 3 days before fluorescent signals degrade	Decrease	No	No	No	No
Hama *et al*. (2011) 9	Sca*l*e	Immersion in Sca*l*eA2 solution (a urea‐based aqueous clearing medium)	2 days (sections) – 2 weeks (whole adult mouse brain)	Unknown	Increase	Limited	No	No to lipophilic dyes 11	No
Ertürk *et al*. (2012) 8	3DISCO	THF to dehydrate tissue; DBE for refractive index matching	2–5 days (whole adult mouse brain)	Days in DBE before fluorescent signals degrade (many months if AlexaFluor is used)	Decrease	Yes 24	No	Yes to CTb 24 No to lipophilic dyes	No
Kuwajima *et al*. (2013) 11	ClearT	Immersion in graded formamide to 95% formamide	~2.5 days (whole mouse brain); 35 mins (1 mm sections)	Formamide is unsuitable for long‐term storage	No change or mild expansion	No	No	Yes	No
Kuwajima *et al*. (2013) 11	ClearT2	Immersion in graded formamide to 50% formamide/20% polyethylene glycol (PEG) solution	~18 h (whole mouse brain); up to 75 mins (1 mm sections)	Formamide is unsuitable for long‐term storage	No change or mild expansion	Yes	No	Yes	No
Chung *et al*. (2013) 13	CLARITY	Formation of hydrogel‐tissue hybrid matrix with acrylamide and bisacrylamide. Clearing of tissue with 4% SDS in boric acid buffer	~2 weeks (whole adult mouse brain)	Many months	Transient increase	Yes	Yes	No to lipophilic dyes 25	Yes
Ke *et al*. (2013) 10	SeeDB	Immersion in graded fructose to saturated fructose with 0.5% *α*‐thioglycerol	About 3 days (embryonic mouse brain)	Up to 1 week in SeeDB solution; longer in PBS	No change	No	No	Yes	No
Susaki *et al*. (2014) 12	CUBIC	Immersion in N,N,N′,N′‐tetrakis(2‐hydroxypropyl)ethylenediamine (25%), Triton X‐100 (wt 15%) and urea (~4 M) for tissue clearing. Then immersion in sucrose (50%), 2,20,20′‐nitrilotriethanol (10%), Triton X‐100 (v/v 0.1%) and urea (~4 M) for refractive index matching	2 weeks (whole mouse brain)	Many months immersed in 20% (w/v) sucrose in PBS, and stored in OCT compound at −80°C	Transient increase	Yes	No	Unknown	No

BABB, benzyl alcohol–benzyl benzoate; CTb, cholera toxin B subunit; DBE, dibenzyl ether; OCT, optimal cutting temperature; PBS, phosphate‐buffered saline; SDS, Sodium dodecyl sulphate; THF, tetrahydrofuran.

CLARITY (an acronym for Clear Lipid‐exchanged Acrylamide‐hybridized Rigid Imaging/ Immunostaining/ in situ‐hybridization‐compatible Tissue hYdrogel) is a novel tissue clearing strategy that aims to overcome the problems mentioned above, by rendering tissue transparent whilst preserving its capability for molecular probing 13. In brief, this technique uses cross‐linking of hydrogel monomers and formaldehyde to functional groups on proteins and nucleic acids. Upon polymerization, a stable hydrogel‐tissue matrix is formed and subsequently, lipids from the tissue are washed off using sodium dodecyl sulphate (SDS) detergent. The resultant hydrogel‐tissue complex can then be immunostained and visualized with confocal or light‐sheet microscopy.

The vast majority of the studies involving CLARITY, including its original description, were designed for rodent brain tissues. However, the presumed requirement of a complicated electrophoretic set‐up, and the complexity and costly nature of the technique have regrettably hindered its use in many laboratories. CLARITY has been applied on human brain tissue 13, 14, 15, but so far only the less myelinated brain tissues of a child 13, 15 or thin 500 *μ*m sections 13, 14 have been visualized. Furthermore, a protocol applicable for a variety of human brain tissue types has not yet been optimized. As a result, we set out to compare methodological differences between mouse, rat and human brain tissues, using passive CLARITY tissue clearing as described by Yang *et al*. 16 and Tomer *et al*. 17. With the potential of CLARITY being applied on human tissue to aid neuropathological investigations, such as the 3D visualization of amyloid‐beta plaques in Alzheimer's brain tissue as reported briefly in one study 14, we also aimed to demonstrate the utility of CLARITY in pathological investigations of *post mortem* Parkinson's disease brain tissues.

## Methods

### CLARITY hydrogel embedding for rodent brain tissue

12‐week‐old C57BL/6 mice and Sprague–Dawley rats weighing between 250–300 g were terminally anaesthetized with sodium pentobarbital (intraperitoneal injection; 120 mg/kg body weight) and transcardially perfused with ice‐cold phosphate‐buffered saline (PBS, pH7.4) and then with hydrogel monomer solution consisting of 4% paraformaldehyde (PFA), 2% or 4% (vol/vol) acrylamide (Bio‐Rad, UK), 0.05% (vol/vol) bis‐acrylamide (Bio‐Rad, UK), 0.25% (wt/vol) VA‐044 photoinitiator (Wako, Alpha‐Labs, Eastleigh, UK) and PBS. Brains were then extracted and immersed in the same solution for 2 days at 4°C. Next, the brains with hydrogel solution were transferred into a 50 ml container and a layer of olive oil was poured on top of the solution with the lid tightly screwed to prevent oxygen inhibition of the subsequent polymerization of the hydrogel by incubating at 37°C for 3 h (or 4 h for 2% acrylamide solution). After removal of excess hydrogel from the hydrogel‐tissue hybrid, brain tissues were left either undissected, bisected into two hemispheres in the case for mouse brains or cut into 3‐mm‐thick coronal sections for rat brains using a rat brain matrix before proceeding to the subsequent clarification step.

### CLARITY hydrogel embedding for human brain tissue

Human brain tissues from the Parkinson's UK Tissue Bank at Imperial College London were used in this study. Both fresh and formalin‐fixed (>4 weeks fixation) tissues from various brain regions were obtained.

Tissue blocks were cut into 0.5–1‐cm‐thick and incubated in hydrogel monomer solution as described above. Fresh brain tissues were incubated for 5–7 days and formalin‐fixed tissues were incubated for 7–10 days (depending on the block thickness) at 4°C until a rubbery‐firm consistency was reached. The tissue blocks with hydrogel solution were then transferred into 50 ml containers and a layer of olive oil was poured on top of the solution, with the lid tightly screwed on to prevent oxygen inhibition of the subsequent polymerization of the hydrogel by incubating at 37°C for 3 h (or 4 h for 2% acrylamide solution). After removal of excess hydrogel from the hydrogel‐tissue hybrid, tissues were then sectioned into 3 mm blocks using a scalpel prior to clearing.

### Passive tissue clearing

A passive clearing protocol, outlined by Tomer and colleagues 17, was adopted in preference to active electrophoretic clearing as described in the original protocol 13. Hydrogel‐embedded samples were washed with SDS clearing solution (4% wt/vol SDS in 1 M boric acid solution, pH8.5; 2 × 24 h at room temperature, RT) to remove remaining hydrogel monomers. Samples were then incubated at 50°C in SDS clearing solution to begin the passive clearing process. Clearing solution was replaced every 2–3 days. Tissue remained in the clearing solution until transparency was achieved.

### Immunostaining on clarified rodent and human brain tissues

After clearing, samples were washed thoroughly with PBS with Triton‐X (PBST; 0.1% vol/vol triton‐X and 0.01% wt/vol sodium azide in PBS) (2 × 24 h at 37°C) to remove residual SDS micelles. Samples were incubated with primary antibody in 15 ml containers with PBST as diluent. Working concentrations for various antibodies used are listed in Table 2. Typically, 1 ml of antibody solution is sufficient to cover a 3‐mm‐thick sample in the container. Tissue samples were incubated at 37°C for 5 days. Primary antibody was washed off with PBST (2 × 24 h at 37°C). Secondary antibody was applied at a concentration of 1:50 and incubated at 37°C for 5 days. A nuclear counterstain, 4′,6‐diamidino‐2‐phenylindole (DAPI; 1:100 from a stock of 1 *μ*g/ml diluted with 1:1 water: dimethyl sulphoxide), can be added at this stage. Tissue was then washed with PBST (2 × 24 h at 37°C) to remove excess secondary antibody.

**Table 2:** Antibodies used in the study

Primary antibodies	Catalogue no.	Company	Host	Clonality	Dilution
Antigen					
Neurofilament (NF, 2F11)	M0762	Dako	Mouse	Monoclonal	1:50–1:100
Ionized calcium‐binding adapter molecule 1 (IBA1)	019‐19741	Wako	Rabbit	Polyclonal	1:50
*α‐*synuclein (*α*‐syn, 42)	610787	Becton Dickson (BD)	Mouse	Monoclonal	1:50
Tyrosine hydroxylase (TH)	AB152	Millipore	Rabbit	Polyclonal	1:50–1:100

Secondary antibodies	Catalogue no.	Company	Host	Fluorophore conjugates	Dilution
Name					
AlexaFluor anti‐goat	A‐11055	Invitrogen	Donkey	488 IgG (H+L)	1:50
AlexaFluor anti‐goat	A‐11057	Invitrogen	Donkey	568 IgG (H+L)	1:50
AlexaFluor anti‐rabbit	A10042	Invitrogen	Donkey	568 IgG (H+L)	1:50
AlexaFluor anti‐mouse	A‐21202	Invitrogen	Donkey	488 IgG (H+L)	1:50
AlexaFluor anti‐rabbit	A‐11011	Invitrogen	Goat	568 IgG (H+L)	1:50
AlexaFluor anti‐mouse	A‐11001	Invitrogen	Goat	488 IgG (H+L)	1:50

### Comparison of various refractive index matching media with human brain tissues

For optimizing tissue refractive index (RI) matching, roughly 1 × 1 × 1 cm^3^ of clarified human cerebellum was each stained in 1:100 dilution of antibodies against neurofilament in 1 ml of PBST at 37°C for 2 days, followed by washing in excess PBST at 37°C for 6 h, and stained with secondary antibody at 1:100 dilution in 1 ml of PBST at 37°C for 2 days. After several washings, the samples were immersed in 5 ml of each compared RI matching formula or cleared as described by various techniques (Table 3).

**Table 3:** Comparison of different refractive index matching solutions

Reagent	Fluorescent signal preservation	Transparency	Time to maximal clearance (3 × 3 × 3 mm^3^ tissue block)	Preparation	Storage	Other remarks
PBST	Good	+	Never	Simple	Indefinitely	N/A
FocusClear	Good	+++	2 h	(Bought)	Cannot be stored	Occasional yellow precipitates or discoloration observed
87% Glycerol	Good	++	Overnight	Simple	Not recommended, slight loss of signal after 2 days	Cheapest and simplest
BABB	Poor	++	3 h	Simple but hazardous	Cannot be stored	Over‐shrinkage
Histodenz‐RIMS	Moderate in humans, good in mice	+++	Overnight	Complex	Up to 6 months for mice, up to 1.5 months for humans	Works best for mice brains
Sorbitol (sRIMS)	Good	+++	Overnight	Complex	Up to 6 months for mice, up to 1.5 months for humans	Take extra care to avoid fungal growth
SeeDB	Moderate	++	6 h	Complex	Up to 1.5 months for humans	Take extra care to avoid fungal growth
Sca*l*eA2	Good	++++	3 h	Simple	Up to 1.5 months for humans	Tissue remains swollen/swells even more; most transparent
THF/DBE	Moderate	++	18 h	Simple but hazardous	Up to 1.5 months for humans	Over‐shrinkage
47% TDE in PBS	Good	++++	3 h	Simple	Up to 1.5 months for humans	Remains slightly swollen
63% TDE in PBS	Good	+++	3 h	Simple	Up to 1.5 months for humans	Shrinks back to original size

BABB, benzyl alcohol–benzyl benzoate; DBE, dibenzyl ether; PBS, phosphate‐buffered saline; PBST, phosphate‐buffered saline with Triton‐X; RIMS, refractive index matching solution; TDE, 2,2′‐Thiodiethanol; THF, tetrahydrofuran.

### Imaging

For imaging of immunostained whole or hemisected mouse brain samples, the CLARITY‐treated brain was incubated in 87% glycerol overnight at room temperature. For immunostained 3‐mm‐thick coronal rat brain or human brain samples, the clarified tissues were incubated in 87% glycerol or Sca*l*eA2 solution for 3 h prior to imaging. Samples were mounted in a 50‐mm diameter standard bottom imaging dish (Ibidi, Germany) with a surrounding ring of Blu‐Tack to prevent sample movement. Samples were placed carefully at the bottom of the dish. RI matching solution was slowly pipetted to the dish, so as not to agitate the sample. It was paramount that the sample remained in contact with the bottom of the dish to ensure the sample was in focus during imaging, due to the short working distance of the objective.

Imaging on CLARITY‐treated samples was performed using a Zeiss LSM‐780 inverted confocal and Zeiss LSM‐710 upright confocal laser scanning microscopes (Carl Zeiss, Oberkochen, Germany) either at the Facility for Imaging by Light Microscopy (FILM) facility in Hammersmith Hospital or at the University of Hong Kong Li Ka Shing Faculty of Medicine Faculty Core Facility. A ×20 objective (W Plan Apochromat DIC M27, numerical aperture 1.0; working distance, 1.7 mm) with laser excitation at 800 nm was used to image the whole mouse brain tissue. To image various human tissues, a ×10 objective (EC Plan‐Neofluar, numerical aperture, 0.3; working distance, 5.2 mm) and ×20 objective (Plan Apochromat DIC, numerical aperture, 0.8; working distance, 0.55 mm) with laser excitation at 405 nm, 488 nm, 543 nm and 594 nm were used. Image capture and processing were performed using the Zen Black (Carl Zeiss, Germany) software. Three‐dimensional rendering and video production were performed on Zen Black (Carl Zeiss, Germany), Volocity (PerkinElmer, MA, USA) and Fiji (Image J, NIH) software.

For the comparison of fluorescent signal intensity after immersion in various RI matching solution, a Leica SP5 inverted confocal microscope with a ×10 objective (HC PL APO CS, numerical aperture, 0.40; working distance 2.2 mm) and laser excitation at 514 nm and 561 nm were used. Image capture was performed using the Leica Application Suite Advanced Fluorescence (LAS AF) software. All imaging settings were the same for each RI‐matched sample. Intensity analysis was done using Fiji macros made by Steve Rothery which is downloadable in http://www3.imperial.ac.uk/imagingfacility/resources/macros-n-scripts, using which intensity profile across the diagonals through the whole image Z‐stacks was analysed, and the overall maximum intensity was found and compared. We did not construct a scoring scheme to quantitatively evaluate each RI matching formula, otherwise many samples would be required yet inaccurate due to intervening variables. Overall, the description of fluorescent signal intensity in Table 3 is based on the intensity evaluation, our overall impression of the samples under the microscope, and our experience with different RI matching formulas, which is sufficient as most histological evaluation focus on morphology rather than quantitative analysis.

### Ethical considerations

The work conducted on human tissue was under ethical approval held by the Parkinson's UK Brain Bank at Imperial College London (Registered charity in England and Wales (258197) and in Scotland (SC037554); Multicentre Research Ethics Committee approval reference number: 07/MRE09/72). Parkinson's UK Brain Bank is an approved Research Tissue Bank by the Wales Research Ethics Committee (Ref. No. 08/MRE09/31+5). Informed consent was obtained prospectively for the use of *post mortem* brain tissues and brain samples were obtained and prepared in accordance to the Wales Research Ethics Committee approved protocols. All animal work in this study was performed at the University of Hong Kong with approval from the Committee on the Use of Live Animals in Teaching and Research (CULATR) in the Laboratory Animal Unit (CULATR reference numbers: 3161‐13 and 3494‐14), a fully accredited unit awarded by the Association for Assessment and Accreditation of Laboratory Animal Care International (AAALAC).

## Results

### Comparison of passive CLARITY clearing speed between rodent and human brain tissues

We performed passive CLARITY tissue clearing as described by Yang *et al*. 16 and Tomer *et al*. 17, on whole mouse and rat brains, and 3‐mm‐thick sections on both rat and human brain tissues.

As transcardial perfusion of hydrogel monomer solution is not possible for human tissue, 0.5–1‐cm‐thick brain tissue sections were processed by immersion fixation in the solution. Long‐term formalin‐fixed brain tissues took 7–10 days as compared with 5–7 days for fresh brain tissue to reach a suitable consistency for subsequent polymerization and clearing steps.

**Figure 1:**
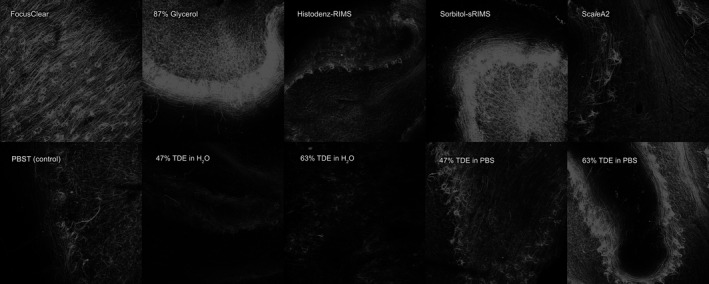
Human cerebellar cortex stained with anti‐neurofilament primary antibody and subsequently AlexaFluor‐568‐conjugated donkey anti‐mouse secondary antibody and imaged using Leica SP5 confocal microscope with a 10× objective, Z‐projections of Z‐stacks 500‐*μ*m thick (z‐stack step size 5 *μ*m), each imaged using the same settings.

In terms of clearing speed, there was a substantial difference between rodent and human brain tissues (Table 4) which may be attributed, at least in part, to the size of the tissue. A whole mouse brain took 21 days (Figure S1**a**–**c**) while a whole rat brain took approximately 60 days to clear. It is important to note, however, that complete transparency of rat brain was not achieved and substantial swelling and yellowing of tissue were seen. Second, comparing clearing speed with tissue of the same thickness (3‐mm thick), human tissue took longer than rodent tissues to reach transparency. For cortical tissue, human tissue took about 3‐ to 4‐times longer than rodent tissue to clear (Figure 1 **d**–**h**). As a result, the total clearing time for a 3‐mm‐thick human brain block was approximately 2 months from dissection (Table 4).

**Table 4:** Comparison of tissue clearing speed between rat and human brain tissue blocks (3‐mm thick)

	Rat	Human (fresh)	Human (formalin‐fixed)
Post‐perfusion immersion fixation	2 days	5–7 days	7–10 days
Washing	2 days	2 days	2 days
Passive thermal clearing	5–10 days (cerebral coronal slices) ~18 days (brainstem)	7–10 days (subcortical blocks) ~40 days (cortical blocks)	~60 days (cortical blocks) Clearing not achieved for brainstem
Washing	2 days	2 days	2 days
Total clearing time from dissection	~11–24 days	~15–51 days	~70 days

Third, different anatomical regions take differing amounts of time to clear, apparently dependent on the degree of myelination. For human tissue, hippocampus and basal ganglia took the shortest time to clear (7–10 days), followed by various cortical regions (~40 days) and brainstem regions caudal to the midbrain failed to clear with passive thermal clearing. Finally, the degree of formalin fixation affects clearing speed. Fresh human brain cortical tissue processed directly in passive clearing took about 40 days to clear which is significantly faster than archival formalin‐fixed cortical tissue which took about 60 days to clear.

The quality of tissue could also be adversely affected by prolonged passive clearing. Notable tissue swelling and some degree of tissue yellowing could be observed for human tissue, perhaps due to residual haemosiderin deposits or lipofuscin‐type pigments present in brain tissue from elderly patients.

We attempted to accelerate the clearing process by reducing acrylamide concentration from 4% to 2% as mentioned in a previous report 16 and we found that rat brain tissue reached transparency faster (5 days as compared to 7 days). However, the clearing speed of the same thickness of human cortical tissue did not alter and tissue became very fragile and collapsed (Figure S1**i**).

### Comparison with different refractive index matching solutions

Refractive index homogenization is an essential step in the CLARITY protocol before imaging. Several more cost‐effective RI matching solutions have been suggested as an alternative to FocusClear^®^ (CelExplorer Labs, Hsinchu, Taiwan) as described in the original protocol. We systematically and experimentally evaluated all available RI matching formula available on clarified mouse and human tissues). In general, for small tissue with shortest diffusion distance less than 2 mm, 3 h of RI matching at room temperature is sufficient, for larger tissues such as whole mouse brain or thick slices up to 1 cm in thickness, overnight RI matching is recommended, and incubation at 37°C can shorten the process to 4 to 5 h.

Based on the refractive index‐matched tissue transparency (Figure S2), fluorescent signal retention (Figure 1), and other practical aspects (Table 3), we found that Sca*l*eA2 rendered tissues most transparent, but it caused substantial tissue swelling.

We then moved on to investigate on 47% 2,2′‐Thidiethanol (TDE), which was the second best in terms of tissue transparency and it shrunk tissues back to its original size, however, a slight yellowish discoloration remained. By observing the effects of diluting TDE in PBS, 1.37 M NaCl (normal saline), 10 mM phosphate buffer (PB), and distilled water on tissue transparency (Figure S3) and fluorescent signal intensity (Figure S4), we were able to reduce the yellow discoloration by diluting 47% TDE in PB instead of the originally proposed PBS, which is recommended for RI matching in human tissues.

### Structural staining of rodent and human tissues

**Figure 2:**
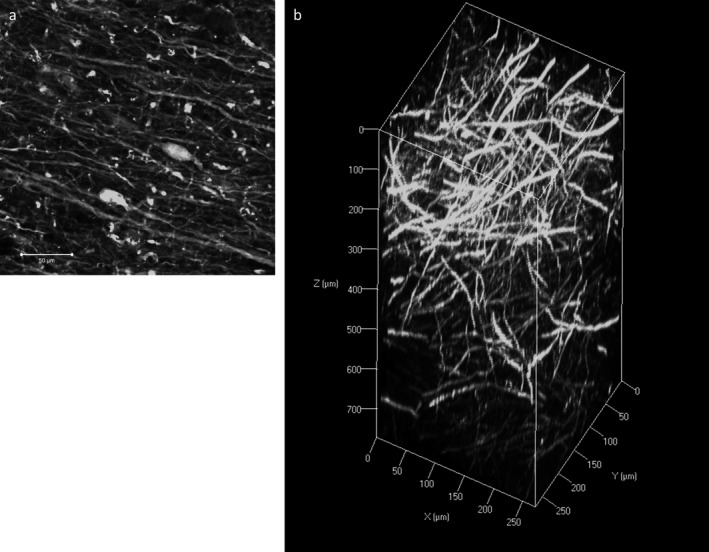
Immunofluorescence with neurofilament (NF) staining on human cortical tissue. **a**: A two‐dimensional image of NF staining showing fine axonal processes and neuronal somas. Scale bar = 50 *μ*m. **b**: Z‐stack image of NF immunostaining on a 3‐mm block of human cortex with an imaging depth to 771.67 *μ*m (z‐stack step size 5.3 *μ*m).

**Figure 3:**
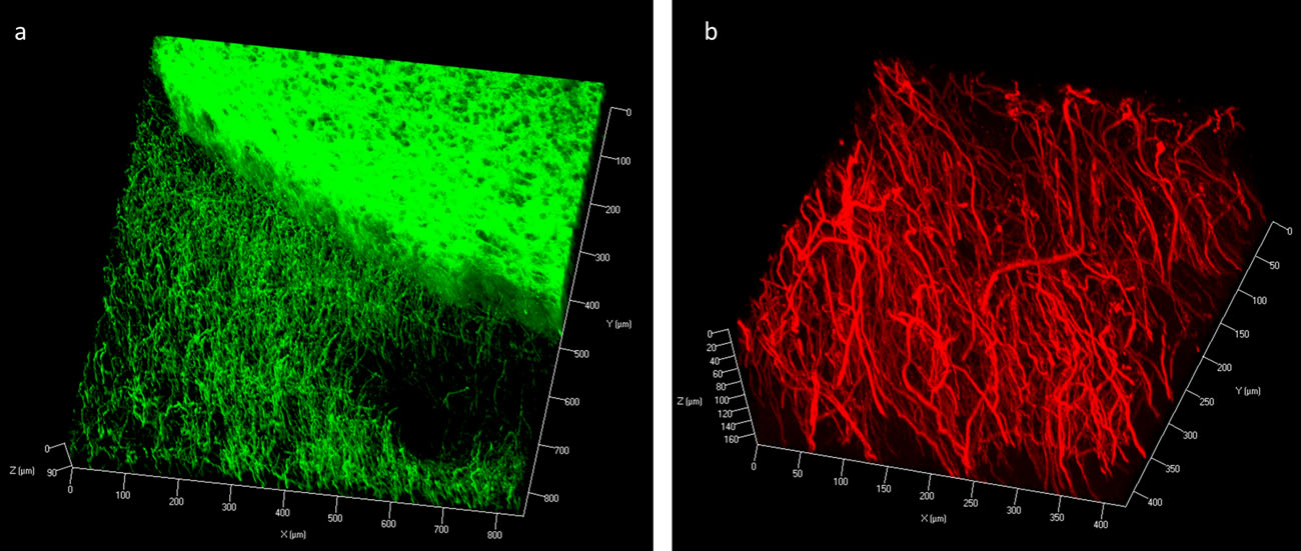
Z‐stack image of Immunofluorescence with tyrosine hydroxylase (TH) staining on rat coronal block showing TH‐positive neuronal processes at the cortex and dense, homogenous staining within the striatum (z‐stack step size 3.3 *μ*m) (**a**). Staining in the human midbrain block showed dense TH‐positive axonal processes (z‐stack step size 1.5 *μ*m) (**b**).

To demonstrate whether the adapted CLARITY technique is translatable from rodent to human tissues for molecular probing, we performed structural immunostaining using anti‐neurofilament (NF) antibodies on human cortical blocks. A detailed linear pattern of axon projections through the depth of the tissue, with neuronal soma was visualized (Figure 2, Video Clip S1) down to a depth of 771.67 *μ*m. However, a decrease in fluorescent signals above 500 *μ*m thickness was noted. Monoaminergic neurones were also visualized with tyrosine hydroxylase (TH) immunostaining. Detailed cortical innervation with dense TH‐positive striatal staining was apparent in clarified rat coronal blocks (Figure 3 **a**) and monoaminergic fibres in the midbrain were also clearly stained in human midbrain tissue (Figure 3 **b**).

**Figure 4:**
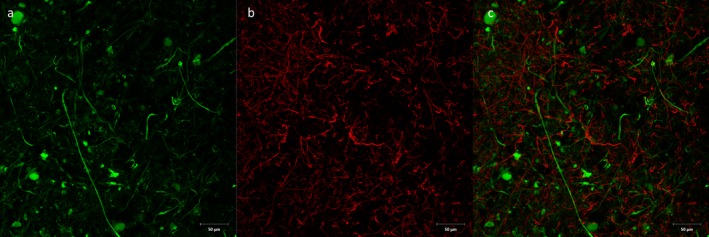
Double immunofluorescence with anti‐neurofilament (**a**, green) and anti‐Iba‐1 (**b**, red) antibodies on human cortical block. **c**: Combined figure showing surveillance network of Iba‐1‐positive microglia around neuronal processes. Scale bar = 50 *μ*m.

We then investigated spatial relationships between neurones and glia using double staining with anti‐NF and anti‐ionized calcium‐binding adapter molecule 1 (IBA1). This revealed the microglial surveillance network surrounding neurones in both mouse and human tissues (Figure 4).

### Pathological investigations in the Parkinson's brain with CLARITY

**Figure 5:**
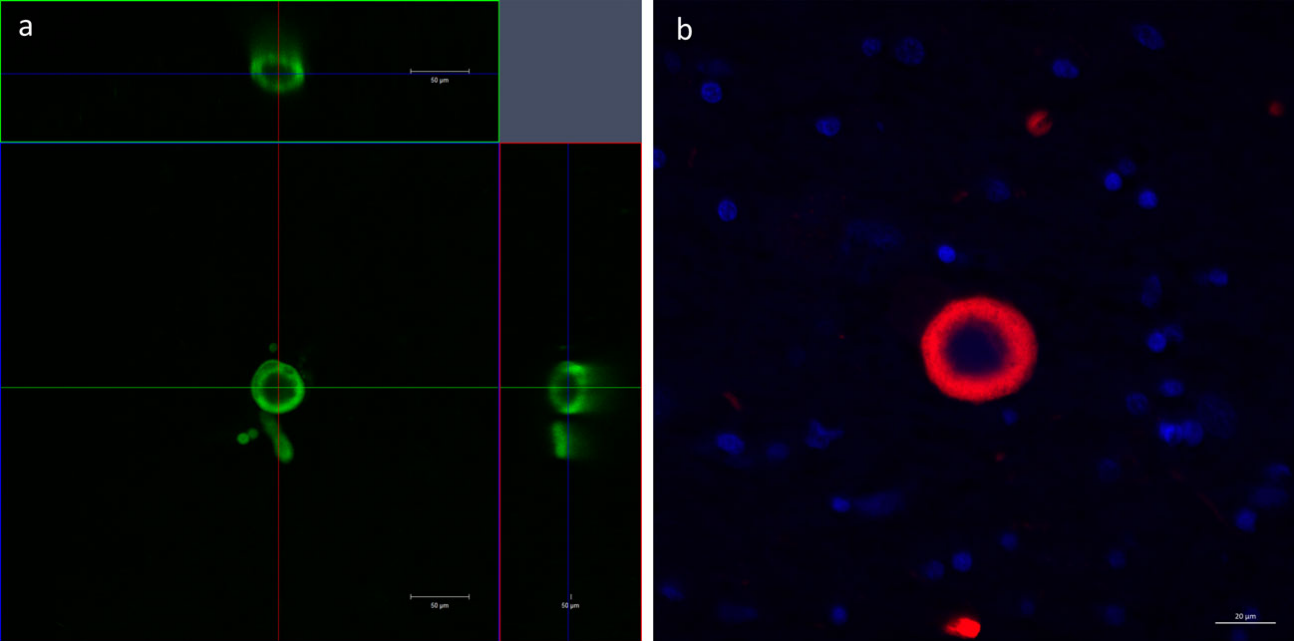
An orthogonal projection of a Lewy body‐like inclusion in a block of cleared tissue containing the nucleus basalis of Meynert in human stained with anti‐*α* SN antibody (green), showing a near‐spherical *α* SN shell of the inclusion in the XY, XZ and YZ planes (Scale bar = 50 *μ*m) (**a**). A confocal Image of a Lewy body‐like inclusion in a standard 7‐*μ*m‐thick section containing the nucleus basalis of Meynert stained with single immunofluorescence with anti‐*α* SN antibody (red) and a nuclear counterstain (DAPI, blue) (Scale bar = 20 *μ*m) (**b**).

**Figure 6:**
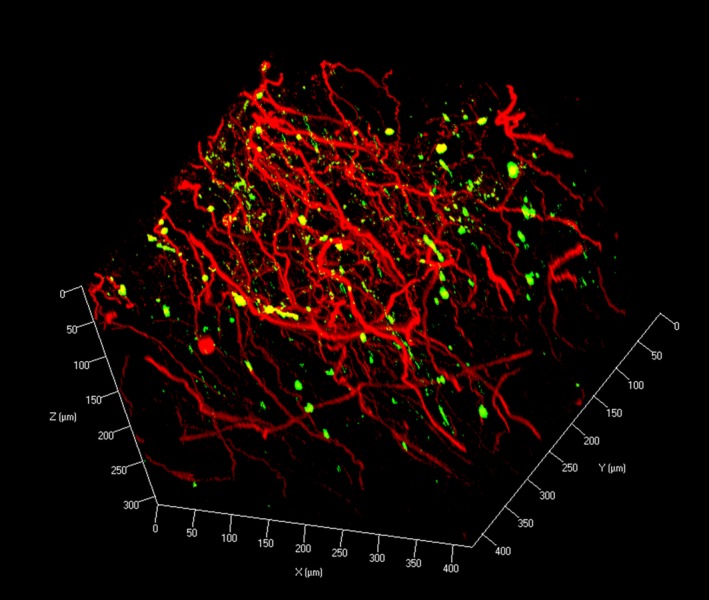
Z‐stack image of double immunofluorescence with anti‐*α* SN (green) and anti‐TH (red) antibodies on human midbrain block (z‐stack step size 1.5 *μ*m).

CLARITY has been shown to be useful for pathological investigations 14. Staining for alpha‐synuclein (αSN) in the nucleus basalis of Meynert of a Parkinson's case revealed characteristic αSN‐immunopositive inclusions that we were able to characterize in three dimensions. In particular, we identified one characteristic brainstem‐type Lewy body using this technique, revealing that a dense, near‐spherical shell of αSN forms the outermost layer of the body as shown in the orthogonal projection image (Figure 5 **a**). This is comparable to Lewy body inclusions seen using immunofluorescence on standard 7‐*μ*m‐thick histological sections (Figure 5 **b**). We were also able to visualize a Lewy neurite as well as a pre‐Lewy body emerging from the side of the larger body (Figure 5 **a**, Video Clip S2). Double immunofluorescence staining with αSN and TH antibodies in the substantia nigra allowed visualization of αSN‐positive inclusions within a dense network of monoaminergic (TH‐positive) fibres. Co‐localization of αSN inclusions with TH‐positive fibres was clearly visualized with CLARITY (Figure 6; Video Clip S3), although not all TH‐positive fibres were found to be immunopositive with αSN as well. In addition, using anti‐TH antibodies with DAPI counterstain revealed sparse TH‐positive fibres within the basal ganglia in a Parkinson's case (Figure S5).

## Discussion

We have demonstrated the passive CLARITY technique, without the need for a complicated electrophoretic set‐up, can be helpful in the investigation of pathology in *post mortem* Parkinson's disease brain. Also, the practical aspects of the passive CLARITY procedure were compared in detail between mouse, rat and human brain tissues. In particular, all currently available RI matching formulations were compared. In addition, the novel visualization of Lewy bodies in 3D from the nucleus basalis of Meynert, and the spatial co‐localization of Lewy pathologies in nigrostriatal monoaminergic fibres highlights the potential of CLARITY in pathological investigations.

When comparing different tissue clearing strategies, CLARITY is often regarded as being expensive and complicated. However, from our work on the rodent and human tissue, we found that this is not necessarily the case. Although the original protocol recommended the use of an electrophoretic set‐up, a subsequent methodology paper 17 actually suggested an advanced CLARITY protocol with passive thermal clearing.

High cost of antibody was thought to be a major concern. However, after a detailed cost analysis with various primary and secondary antibodies used in this study, it was shown this cost was not significant even with the use of maximum concentration as suggested in the original CLARITY protocol (1:50 dilution). In fact, for the same thickness of tissue, the use of traditional serial sectioning through the entire tissue block would potentially consume a greater amount of antibodies. Assuming sections are cut at 10‐*μ*m thick, this would yield 300 sections for a 3‐mm‐thick block. Using an optimized dilution for immunohistochemistry on tissue sections, this could cost up to two‐ to three‐times more than CLARITY‐treated tissue blocks.

The use of SDS in CLARITY has been criticized for its potential for causing damage to the tissue. However, we did not observe any significant structural or morphological differences after tissue clearing in either rodent or human tissue. Deisseroth's group has demonstrated protein loss with CLARITY at 8% as compared to 65% when a hydrogel‐tissue hybrid was not used for tissue clearing 13. For a 2‐mm‐thick tissue cube, we found a comparable 13% protein loss in human brain clearing (unpublished observation). This indicates the possibility of the formation of a hydrogel matrix which may help protect the tissue from damage by SDS. Furthermore, SDS has been traditionally used as an antigen retrieval agent for immunohistochemistry, hence, the use of SDS in tissue clearing could be beneficial in aiding exposure of several antigens for molecular tagging.

An essential step of CLARITY is RI matching. According to Chung and colleagues (2013), FocusClear^®^ works best as its RI matches that of the hydrogel‐tissue matrix (~1.45). However, the use of this commercially available but expensive reagent was frequently cited to be a prohibitive factor for this technique. We and others have found the use of FocusClear unnecessary and 47% TDE was a more cost‐effective option for human brain tissues. In our hands, in terms of optical transparency and best preservation of fluorescent signals, Histodenz‐RI matching solution (RIMS) worked well in transgenic mouse tissues but not for human ones, which highlights the differences between human and mouse tissues. However, the principles behind this phenomenon remained to be explored. We observed that the amount of yellow discoloration in RI‐matched tissues roughly correlates with the amount of shrinkage, which in turn depends on the osmotic strength of the RI matching solution. We tested this idea by diluting TDE in PB, instead of the original formulation using PBS, for RI matching. Indeed, removing the saline component of PBS reduces yellow discolorations and improves transparency (Figure S3); RI matching with 47% TDE in water results in remarkable transparency, but also greatly reduces fluorescence intensity (Figure S4), presumably due to pH fluctuations as the fluorescent dyes are pH‐sensitive. As yellow discoloration can be prominent in large human tissue samples, our observation and deduction should prompt future development of a better buffering agent with lower osmotic strength.

Currently, there are only three published reports involving CLARITY on human brain tissues (Table 5) 13, 14, 15 and in each study the tissue was cleared and visualized with slightly different conditions. The original method paper processed 500 *μ*m blocks of tissue from a 7‐year‐old autistic patient, showing the 3D rendering of neuronal projections, as well as a specialized, atypical laddering pattern of neurones, characteristic of neurones from animals with autism‐like behaviour 13. 500‐*μ*m‐thick brain samples from Alzheimer's disease patients, immunostained for tau and amyloid‐beta, revealed the 3D structures of plaques and tangles and the interaction between the two pathologies. In addition, this group tried other clearing techniques (Sca*l*e, SeeDB, 3DISCO), but found that CLARITY gave the best results 14. Most recently, sections from a patient with hemimegalencephaly have been CLARITY‐processed, allowing for three‐dimensional tracing of GABA‐ergic interneurones in a 2‐mm section of tissue 15.

**Table 5:** Current studies with CLARITY involving the use of human brain tissues

Reference	Cases	Sample	Tissue type	Thickness (*μ*m)	Clearing time	Immunostaining time	Antibodies used	Refractive index matching medium
Chung *et al*. (2013) 13	7‐year old male with autism; 10‐year old male control	Frontal cortex	Formalin‐fixed	500	1 day (active clearing)	3 days	MBP, TH, PV, NF	FocusClear
Ando *et al*. (2014) 14	5 Alzheimer's disease 2 controls	Frontal cortex	Formalin‐fixed	500	2 weeks (37°C, passive clearing)	Not reported	Abeta, tau, NF	Sca*l*eA2
Costantini *et al*. (2015) 15	1 child with hemi‐megalencephaly	Cortex	Formalin‐fixed	2000	2 weeks (37°C, passive clearing)	6 days	PV, GFAP	47% TDE

GFAP, Glial fibrillary acidic protein; MBP, myelin basic protein; NF, neurofilament; PV, Parvalbumin; TDE, 2,2′‐Thiodiethanol; TH, tyrosine hydroxylase.

We expanded on these studies by clarifying different regions of the brain in addition to cortical blocks. Although the clearing speed for our samples were slightly longer (7–60 days) than that in other studies (1–14 days), this could be due to the thicker samples we used for CLARITY (3 mm *vs*. 0.5‐ to 2‐mm‐thick tissues) and the more heavily myelinated tissue from elderly patients we used compared with children's brain 13, 15. Furthermore, we have shown that the CLARITY technique can be used with unfixed as well as formalin‐fixed human brain tissue.

Interestingly, none of the studies involving the human brain, including this study, was successful in imaging cleared tissues beyond 1 mm in depth. Imaging depth of CLARITY appears to be limited by several factors. First, as mentioned above, different RI matching reagent has varying effect on optical transparency and preservation of fluorescence signal. Currently available reagents may still not be optimal. Second, optical properties of the objectives of microscopes, in particular the working distance, is a physical restraint to the size of the piece of tissue able to be processed with CLARITY. However, some of the long‐working distance objectives nowadays can image tissue up to 12 mm in depth. Third, the depth of penetration of antibodies is perhaps the biggest limitation currently. Although the exact physical diffusion limit of antibodies is yet to be determined, it appeared that some antibodies (e.g. anti‐TH) could penetrate a greater depth than others targeting antigens which are more abundantly expressed (e.g. IBA‐1). We hypothesize this is due to “overcrowding” of antibodies at the surface for densely expressed antigens which prevents further penetration into the tissue. Further work is therefore needed to identify the practical limit and optimal parameters (including dilution factor and incubation times) for antibody penetration targeting different antigens to allow thorough investigation of large anatomical structures in the human brain.

Even though CLARITY cannot yet be used on a large human brain blocks for connectomic studies, spatial interaction between different proteins could be clearly visualized. In this study, we presented the spatial co‐localization of Lewy pathology and monoaminergic (TH‐positive) fibres in the midbrain of a *post mortem* human brain. This study is the first to visualize the three‐dimensional structure of a Lewy body, supplementing findings from a previous study 18 which gathered this information by serial sectioning and computerized reconstruction of brain tissue. Using CLARITY, we validated that a Lewy body is composed of a spherical or near‐spherical αSN shell simply with optical sectioning with confocal microscopy.

CLARITY is a powerful technique with great potential, not only for neuroscience research as it is also applicable for other tissue types 19. However, this technique is still in its infancy and some inherent limitations remain. For example the possibility of antigen re‐probing was described in the original protocol 13 but so far there have been no further reports confirming the success of this approach. We attempted this on human brain tissue but some residual fluorescent signals were present (unpublished observation). In addition, tissue swelling after active or passive clearing in CLARITY was reported to be transient. This has yet to be validated as certain morphological and structural changes, e.g. breaking of blood–brain barrier integrity, could be produced artificially. In addition, even though CLARITY has great molecular probing capacity, antibodies which normally require complicated antigen retrieval procedures might still pose compatibility issues with CLARITY.

Nevertheless, the CLARITY technique is constantly evolving and since its first description, several groups have improved on the “active” electrophoretic tissue clearing, including the use of 3D printing of a CLARITY electrophoretic clearing chamber 20, optimization of active clearing on various tissue organ 19 as well as the use of the electrophoretic apparatus to aid penetration of antibodies to visualize deeper into the tissue 21. To improve the passive clearing procedure, thickness of the tissue 22 and the composition of the hydrogel monomer cocktail has been altered 16, 23. In addition, the introduction of CLARITY‐optimized optics as well as light sheet microscopy technique 17 will hopefully further increase the utility of this technique. Our future work will include the full optimization of the CLARITY protocol for the use on human brain tissue as this technique has limitless potential in contributing to our understanding of human neurological conditions.

## Author contributions

AKLL, RCCC and SMG designed and conceived the study. AKLL and MEDH drafted and revised the manuscript. AKLL, MEDH and HML carried out the human CLARITY experimental work for the study. AKLL and OTWN carried out the rodent CLARITY experimental work for the study. JDeF provided technical support for the study. RKBP, GTCW, RCCC and SMG supervised the research. All authors read, reviewed and edited the final manuscript.

## Supporting information


**Figure S1.** Comparison of clearing speed between different tissues. **a**–**c**: Clearing of a whole mouse brain to transparency in 21 days. **d**–**f**: Clearing of a 3‐mm block of rat brain to transparency in 10 days. **g**,** h**: Clearing of a 3‐mm block of human cortical tissue to transparency in 39 days. **i**: human brain tissue cross‐linked with 2% instead of 4% acrylamide does not improve clearing speed. Tissue integrity is also considerably worse than that with 4% acrylamide.

**Figure S2.** 1 × 1 × 1 cm^3^ of human cerebellum stained with anti‐neurofilament and refractive index‐matched for 28 h at room temperature in different formula/techniques, upper row from left to right: FocusClear, 87% Glycerol, Histodenz‐RIMS, Sorbitol (sRIMS), ScaleA2, SeeDB, PBST (control), 47% TDE in water, 63% TDE in water, 47% TDE in PBS, 63% TDE in PBS; lower left: clearing by dehydration (methanol series)‐BABB method; lower right: clearing by dehydration (THF series)‐DBE method.

**Figure S3.** 1 × 1 × 1 cm^3^ of human cerebellum stained with anti‐neurofilament and RI‐matched for 28 h at room temperature in 47% TDE with different diluents, from left to right: in PBS, in 1.37 M NaCl, in 10 mM phosphate buffer, in water.

**Figure S4.** Z‐projections of samples in Figure S3 RI‐matched in 47% TDE diluted in different medium. From left to right: in PBS, in 1.37 M NaCl, in 10 mM phosphate buffer, in water.

**Figure S5.** A 3 × 3 tiled Z‐projection (z‐stack depth = 419.95 *μ*m, step size = 2.295 *μ*m) of a basal ganglia section stained using anti‐TH antibody with a nuclear counterstain 4′,6‐diamidino‐2‐phenylindole (DAPI), revealing the sparse monoaminergic fibres in a Parkinson's case. Scale bar = 80 *μ*m.

**Video Clip S1.** Three‐dimensional visualization of neurofilament immunohistochemical staining in *post mortem* human cortical tissue. A 3‐mm block of human cortex was immunostained using anti‐neurofilament antibody (green). Stained tissue was visualized using a 10× objective (EC Plan‐Neofluar, numerical aperture, 0.3; working distance, 5.2 mm) with an imaging depth to 771.67 *μ*m (z‐stack step size 5.3 *μ*m).

**Video Clip S2.** Three‐dimensional visualization of a Lewy body in the human nucleus basalis of Meynert. A 3‐mm block of human nucleus basalis of Meynert was immunostained using anti‐alpha‐synuclein antibody (green). Stained tissue was visualized using a 20× objective (Plan Apochromat DIC, numerical aperture, 0.8; working distance, 0.55 mm; z‐stack step size 1.1 *μ*m).

**Video Clip S3.** Three‐dimensional visualization of monoaminergic fibres and Lewy pathologies in the *post mortem* human midbrain tissue. A 3‐mm block of human midbrain tissue was immunostained using anti‐alpha‐synuclein antibody (green) and tyrosine hydroxylase (red). Stained tissue was visualized using a 20× objective (Plan Apochromat DIC, numerical aperture, 0.8; working distance, 0.55 mm) with an imaging depth to 312.62 *μ*m (z‐stack step size 1.5 *μ*m).
