# Supplementary figures and images for: Bringing CLARITY to the human brain: visualization of Lewy pathology in three dimensions
Source: Neuropathol Appl Neurobiol. 2015 Dec 7;42(6):573–87. doi: 10.1111/nan.12293 (PMC5053282; doi:10.1111/nan.12293)

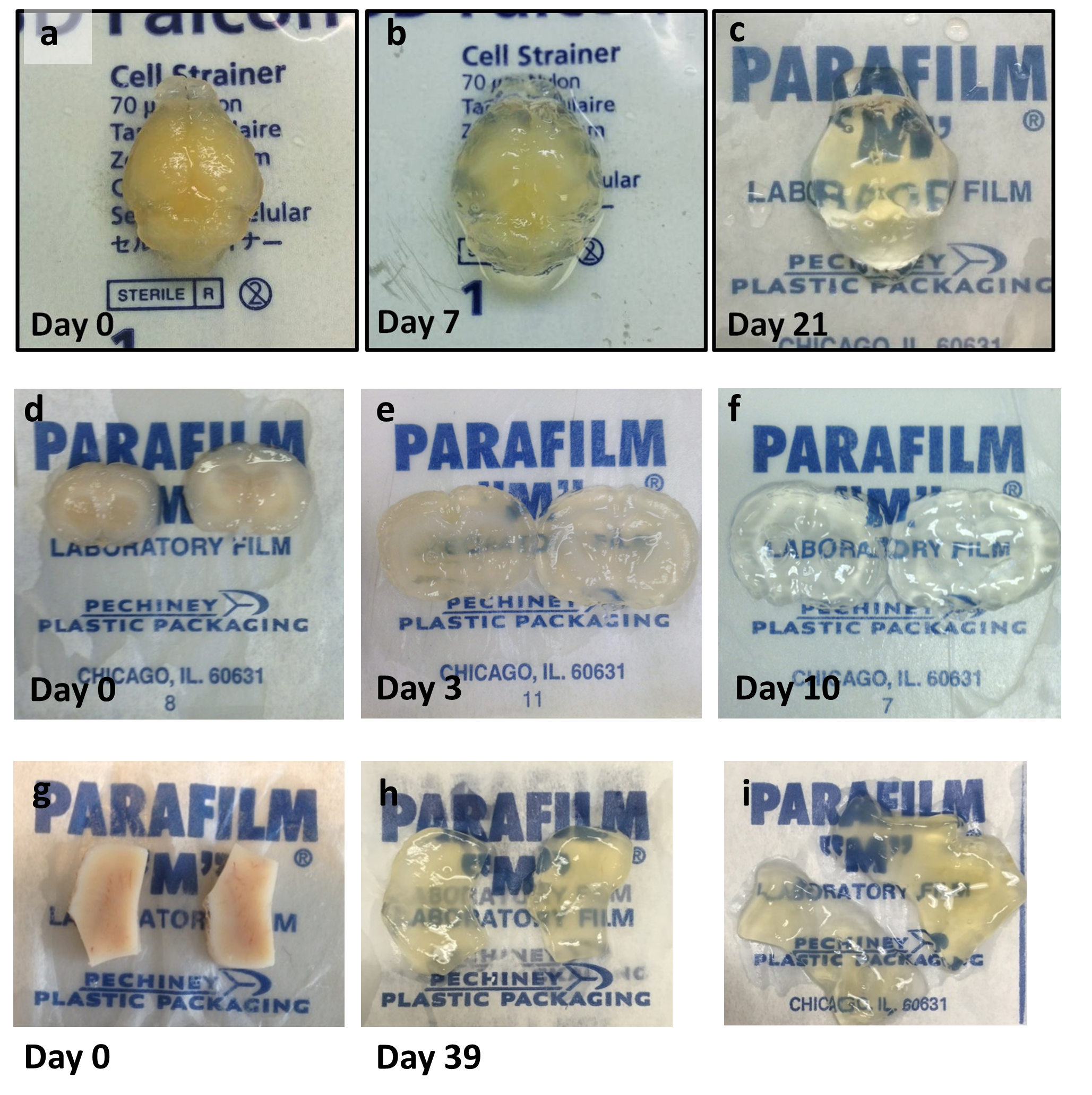

Supplement: Supplementary file 1 — Figure S1. Comparison of clearing speed between different tissues. a–c: Clearing of a whole mouse brain to transparency in 21 days. d–f: Clearing of a 3‐mm block of rat brain to transparency in 10 days. g, h: Clearing of a 3‐mm block of human cortical tissue to transparency in 39 days. i: human brain tissue cross‐linked with 2% instead of 4% acrylamide does not improve clearing speed. Tissue integrity is also considerably worse than that with 4% acrylamide. [file NAN-42-573-s001.tif]

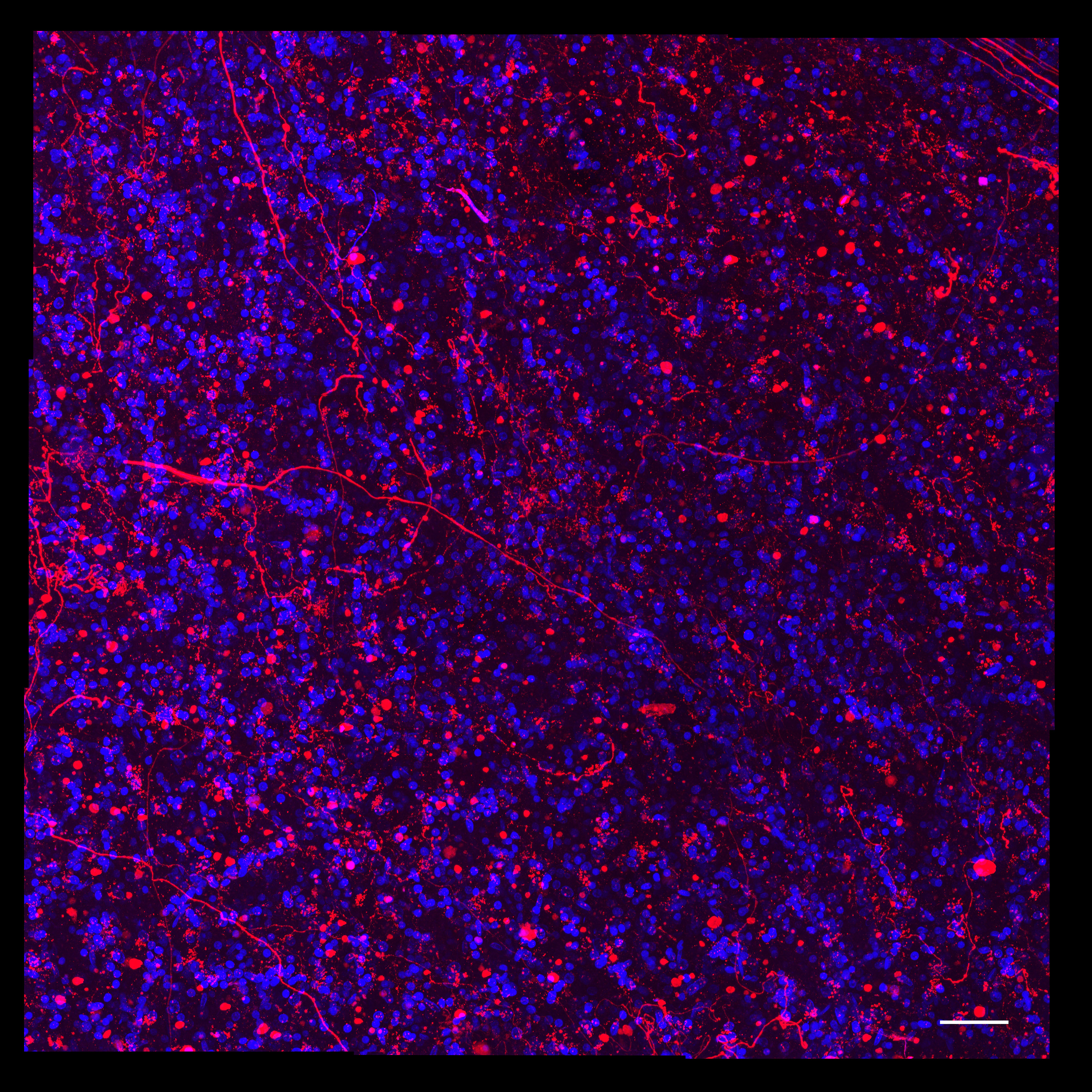

Supplement: Supplementary file 5 — Figure S5. A 3 × 3 tiled Z‐projection (z‐stack depth = 419.95 μm, step size = 2.295 μm) of a basal ganglia section stained using anti‐TH antibody with a nuclear counterstain 4′,6‐diamidino‐2‐phenylindole (DAPI), revealing the sparse monoaminergic fibres in a Parkinson's case. Scale bar = 80 μm. [file NAN-42-573-s005.jpg]
